# Advances in Nanohybrid Hydrogels for Wound Healing: From Functional Mechanisms to Translational Prospects

**DOI:** 10.3390/gels11070483

**Published:** 2025-06-23

**Authors:** Yunfei Mo, Tao Zhou, Weichang Li, Yuqing Niu, Chialin Sheu

**Affiliations:** 1 School of Life and Health Technology, Dongguan University of Technology, Dongguan 523808, China; 2 Guangdong Provincial Key Laboratory of Stomatology, Guanghua School of Stomatology, Sun Yat-sen University, Guangzhou 510055, China

**Keywords:** nanohybrid hydrogel, wound healing, responsive biomaterials, antibacterial hydrogel, angiogenesis

## Abstract

Chronic wounds, such as diabetic ulcers and pressure injuries, remain a major global health burden, affecting over 40 million people worldwide and imposing significant socioeconomic strain. Hydrogel-based wound dressings have gained clinical attention for their ability to maintain moisture, mimic the extracellular matrix, and support tissue regeneration. However, traditional hydrogels often lack the mechanical robustness, antimicrobial efficacy, and dynamic responsiveness needed to treat complex wound environments effectively. To address these limitations, nanohybrid hydrogels, composite systems that integrate functional nanomaterials into hydrogel matrices, have emerged as intelligent platforms for advanced wound care. These systems enable multifunctional therapeutic action, including antibacterial activity, antioxidant regulation, angiogenesis promotion, immune modulation, and stimuli-responsive drug delivery. This review synthesizes recent advances in nanohybrid hydrogel design, beginning with an overview of traditional polymeric systems and their constraints. We categorize functional mechanisms according to biological targets and classify nanohybrid architectures by material type, including metal-based nanoparticles, nanozymes, carbon-based nanomaterials, polymeric nanogels, and metal–organic frameworks. Representative studies are summarized in a comparative table, and challenges related to biosafety, clinical translation, and design optimization are discussed. Nanohybrid hydrogels represent a rapidly evolving frontier in wound care, offering bioresponsive, multifunctional platforms with the potential to transform chronic wound management.

## 1. Introduction

Wound healing is a complex and tightly regulated biological process involving hemostasis, inflammation, proliferation, and tissue remodeling [1]. However, in conditions such as diabetes, vascular insufficiency, and chronic infection, wounds may fail to progress through the normal stages of healing [2]. Chronic wounds are estimated to affect over 40 million people worldwide, with rising prevalence driven by aging populations, increased rates of diabetes, and antibiotic resistance [3]. These non-healing wounds often require long-term management and are associated with high risks of infection, limb amputation, and recurrence [4]. In high-income countries, they account for up to 5% of total healthcare expenditures, highlighting their profound socioeconomic and clinical impact.

Hydrogel-based dressings have emerged as a cornerstone in modern wound care due to their biocompatibility, high water content, and ability to maintain a moist healing environment [5]. These soft, polymeric networks mimic the physical properties of native extracellular matrix (ECM), promote cellular infiltration, and support the exchange of nutrients and gases [6,7]. A recent meta-analysis of 39 clinical trials, encompassing more than 1700 patients, demonstrated that hydrogel dressings significantly improved wound closure rates and reduced healing time compared to conventional treatments [8]. However, conventional hydrogel systems often fall short in treating complex or chronic wounds, largely due to their poor mechanical durability, limited antimicrobial activity, and inability to respond dynamically to the wound microenvironment [9,10].

To address these limitations, recent research has focused on the development of nanohybrid hydrogels, multifunctional composites that integrate nanomaterials into hydrogel matrices to create intelligent, responsive wound dressings [11,12]. These hybrid systems enable active therapeutic functions such as sustained antimicrobial release, reactive oxygen species (ROS) scavenging, angiogenesis promotion, and immune modulation [13,14,15,16]. A notable contribution by Zhou et al. (2023) demonstrated a platinum nanozyme-based double-network hydrogel capable of orchestrating stage-specific enzymatic functions [17]. Through sequential activation of peroxidase-, catalase-, and glucose oxidase-like activity, the hydrogel adapted to different phases of wound healing, alleviated oxidative stress, and accelerated tissue regeneration in diabetic wound models. This work illustrates the evolution of hydrogel design toward bioresponsive, multifunctional platforms tailored to the multifaceted pathology of chronic wounds.

**Scheme 1:**
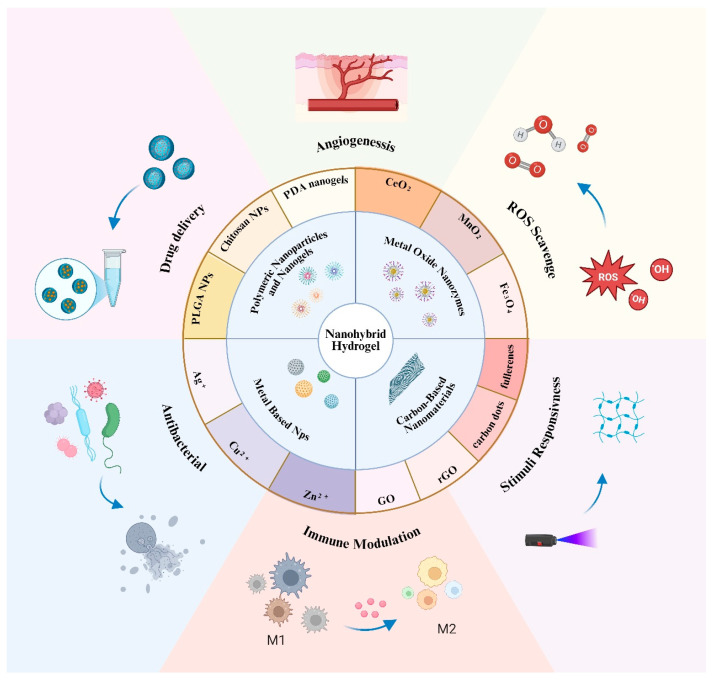
Construction of nanohybrid hydrogel and its application in wounds.

This review presents a comprehensive synthesis of recent advances in nanohybrid hydrogel systems for wound healing, with a focus on both material design and biological function (Scheme 1). We begin by examining the polymeric foundations of traditional hydrogels-highlighting the roles of natural and synthetic polymers, as well as their structural and functional limitations. We then introduce the design principles and therapeutic mechanisms of nanohybrid hydrogels, organized by key functional advantages including antibacterial activity, antioxidant and redox regulation, angiogenesis promotion, immune modulation, and stimuli-responsiveness. Further, we classify nanohybrid systems based on nanomaterial type, analyzing the unique contributions of metal-based nanoparticles, nanozymes, carbon-based nanomaterials, polymeric nanostructures, and metal–organic frameworks. Finally, we summarize representative systems in tabular format and discuss outstanding challenges and future prospects in achieving clinically translatable, intelligent hydrogel therapies.

## 2. Traditional Hydrogels in Wound Healing

### 2.1. Introduction to Traditional Hydrogels

Hydrogels are three-dimensional, crosslinked polymer networks capable of absorbing large volumes of water, often comprising more than 90% of their total mass. Their high water content, tunable mechanical properties, and soft consistency allow them to closely mimic the ECM, making them particularly attractive for biomedical applications such as wound healing [18,19]. By maintaining a moist environment, hydrogels support epithelialization, reduce pain, facilitate autolytic debridement, and protect against secondary infections [20,21]. Moreover, they permit gas exchange while absorbing wound exudate, thereby promoting tissue regeneration in a minimally invasive manner [22]. Since their introduction in the mid-20th century, hydrogel dressings—commercialized in products such as Intrasit^™^, Tegage^™^, and NuGe^™^—have become a mainstay in clinical wound care due to their biocompatibility, ease of use, and capacity for incorporating therapeutic agents [23]. Their success is largely attributed to their biocompatibility, ease of application, and ability to serve as carriers for drugs or growth factors. However, the performance of hydrogels varies widely based on the underlying polymer composition, which may be derived from natural or synthetic sources [10,24]. The following sections discuss these materials in detail, highlighting their biological functionality and material limitations.

In addition to their internal structural composition, the surface coating behavior of hydrogels significantly impacts their wound healing functionality. Properties such as conformal contact with wound beds, surface adhesiveness, and barrier integrity determine how effectively the hydrogel adheres, prevents infection, and promotes re-epithelialization. Functional coatings—especially those incorporating antimicrobial or bioadhesive components—can improve wound coverage, drug retention, and cellular interactions [25,26,27,28]. These surface characteristics thus represent an important design consideration for optimizing clinical outcomes. The following sections discuss these materials in detail, highlighting their biological functionality and material limitations.

### 2.2. Common Materials Used in Traditional Hydrogels

Traditional hydrogels are primarily fabricated from either naturally derived or synthetic polymers, each offering distinct advantages in biocompatibility, degradation behavior, mechanical performance, and application flexibility. The polymer composition governs the hydrogel’s mechanical integrity, fluid absorption, degradation kinetics, and interaction with the wound microenvironment, ultimately shaping its therapeutic outcomes.

#### 2.2.1. Natural Polymer-Based Hydrogels

Naturally derived polymers have long served as foundational materials for hydrogel wound dressings due to their excellent biocompatibility, biodegradability, and structural resemblance to native ECM components. These biopolymers provide supportive microenvironments that facilitate cell adhesion, migration, and proliferation-critical steps in tissue repair.

**Figure 1:**
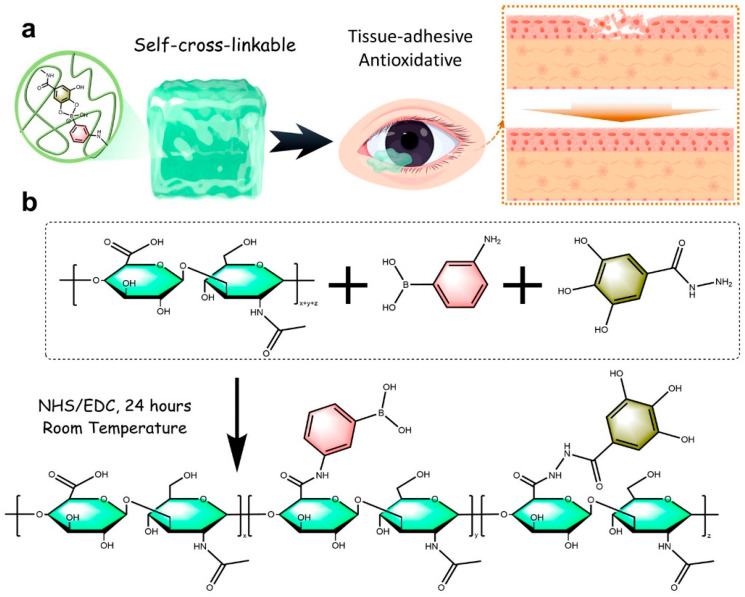
Schematic representations of (**a**) the HA-GA-PBA hydrogel for corneal epithelial wound healing, exhibiting self-crosslinkable, tissue-adhesive, and antioxidative properties; (**b**) the synthetic route for HA functionalized with gallol and phenylboronic acid groups [33]. Copyright © 2025, Elsevier.

**Hyaluronic acid (HA)** is a glycosaminoglycan abundantly present in the skin and connective tissues. Known for its hydrophilicity and interaction with cluster of differentiation 44 (CD44) receptors, HA contributes to inflammation modulation and angiogenesis regulation, while also facilitating moisture retention and granulation tissue formation [29,30,31]. However, native HA suffers from rapid enzymatic degradation and lacks sufficient mechanical strength [32]. To address these limitations, Pan et al. (2025) developed a dual-functionalized HA hydrogel (HA-GA-PBA), incorporating gallol and phenylboronic acid moieties to enable dynamic covalent crosslinking, ROS scavenging, and tissue adhesion (Figure 1) [33]. In vivo application in a mouse corneal wound model demonstrated enhanced epithelial regeneration, reduced inflammatory cytokine expression, and minimized scar formation, highlighting the adaptability of engineered HA systems for targeted wound settings.

**Figure 2:**
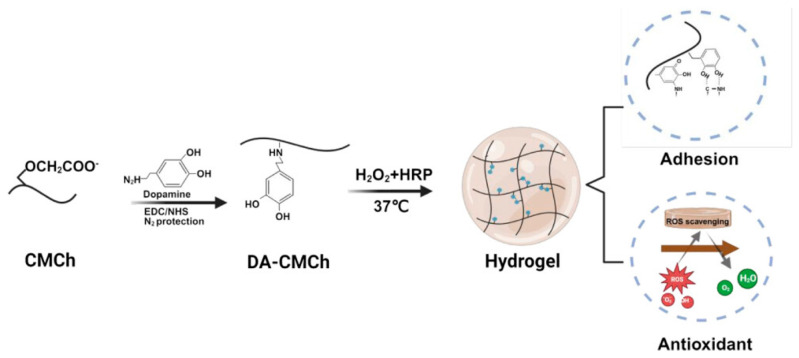
A hydrogel dressing combining in situ-forming, good tissue adhesiveness, and excellent antioxidant capabilities was developed by simply conjugating dopamine onto carboxymethyl chitosan [44]. Copyright © 2025, American Chemical Society.

**Chitosan**, derived from chitin via deacetylation, is widely valued for its intrinsic antibacterial activity, hemostatic capability, and ability to stimulate fibroblast proliferation [34,35,36]. Despite these advantages, its clinical use is hindered by poor solubility under physiological pH and brittle mechanical characteristics [37,38,39,40,41,42,43]. Luo et al. (2025) addressed these challenges by conjugating dopamine to carboxymethyl chitosan (CMCh) and initiating enzymatic crosslinking via horseradish peroxidase and hydrogen peroxide (Figure 2) [44]. The resulting hydrogel displayed strong tissue adhesion, significant antioxidant activity, and enhanced fibroblast proliferation, accelerating wound closure in a rat full-thickness skin injury model [45].

**Figure 3:**
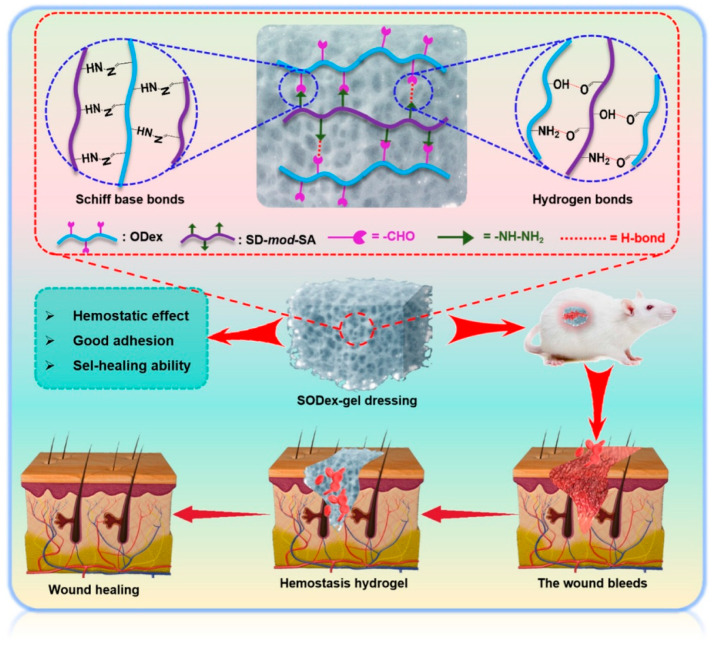
Schematic illustration of the formation of the hydrogel dressing (SODex-Gel), presenting its action characteristics and wound healing ability [51]. Copyright © 2025, American Chemical Society.

**Alginate**, a polysaccharide extracted from brown seaweed, gels readily in the presence of divalent cations such as Ca^2+^, offering excellent exudate absorbency and biocompatibility [46,47,48]. However, its ionic crosslinked network is mechanically unstable under fluctuating wound conditions, and its adhesion to tissue is poor [49,50]. Zhao et al. (2025) addressed these limitations by developing a self-healing hydrogel using oxidized dextran and succinic dihydrazide-modified sodium alginate (SD-mod-SA) (Figure 3) [51]. The hydrogel exhibited high oxygen permeability, strong tissue adhesion, and pro-angiogenic activity, significantly improving burn wound healing in murine models.

**Figure 4:**
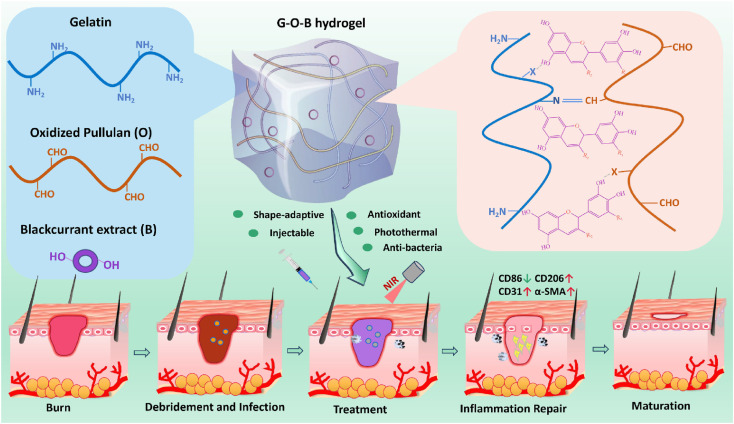
Schematic of the G-O-B hydrogel composed of gelatin, oxidized pullulan, and blackcurrant extract, exhibiting photothermal, antioxidant, and antibacterial properties for infected burn wound healing [57]. Copyright © 2024, Elsevier.

**Gelatin**, a hydrolyzed form of collagen, retains Arg-Gly-Asp (RGD) motifs that enhance cell adhesion and is frequently used in hydrogel systems due to its biocompatibility and ease of modification [52,53,54]. Compared to native collagen, gelatin hydrogels are more processable, but they remain prone to thermal instability and enzymatic degradation [55,56]. To enhance its functionality, Yu et al. (2024) formulated a multifunctional gelatin-based hydrogel incorporating oxidized pullulan and blackcurrant extract (Figure 4) [57]. This G-O-B hydrogel exhibited robust self-healing, photothermal antimicrobial effects, and ROS scavenging capacity. In a deep burn model, it promoted angiogenesis, accelerated wound contraction, and modulated inflammation, demonstrating its potential in managing infected or chronic wounds.

Natural polymer-based hydrogels continue to form a critical backbone in wound dressing design due to their tunable bioactivity, degradability, and capacity for biological signaling. Through chemical modification and blending strategies, their functional range has been expanded to accommodate diverse clinical applications while maintaining favorable cytocompatibility.

#### 2.2.2. Synthetic Polymer-Based Hydrogels

Synthetic polymers have emerged as vital components in hydrogel design due to their high structural tunability, consistent quality, and adaptability for scalable production. Unlike natural polymers, which may vary in bioactivity and degrade unpredictably, synthetic systems allow for precise control over crosslinking density, degradation rate, and mechanical strength. These features make them attractive platforms for tailoring hydrogel dressings to meet the evolving needs of different wound environments, including chronic, inflamed, or irregularly shaped wounds [58,59,60,61,62].

**Figure 5:**
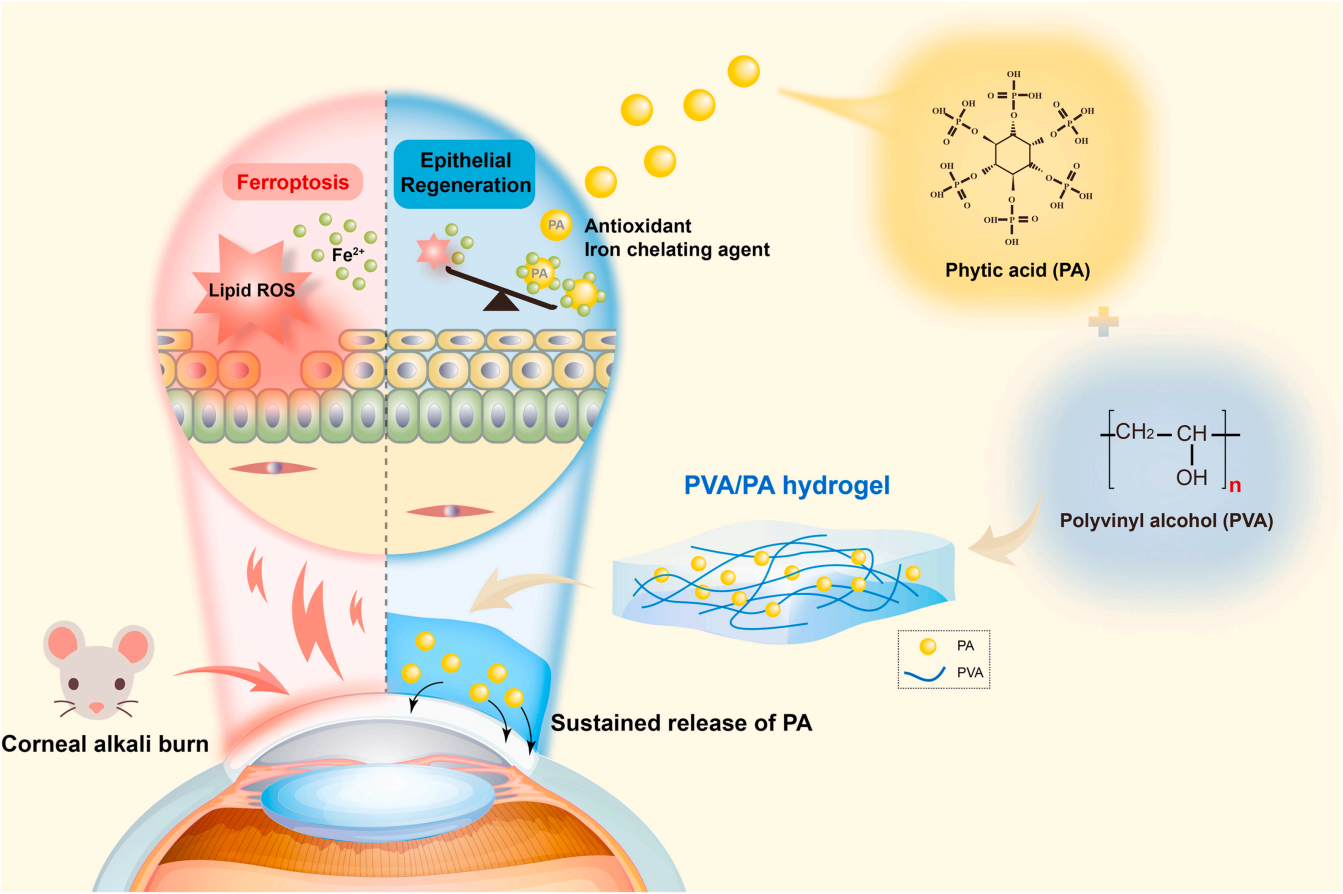
Schematic illustration of the phytic acid-loaded PVA hydrogel designed for corneal epithelial wound healing [67]. Copyright © 2024, Elsevier.

**Polyvinyl alcohol (PVA)** is a water-soluble synthetic polymer known for its excellent film-forming capacity, high mechanical strength, and ease of chemical crosslinking via hydroxyl groups [63,64,65]. Its rheological tunability, moisture retention, and transparency make it well suited for applications in epithelial wound care [66]. Gong et al. (2024) developed a PVA-based hydrogel incorporating phytic acid (PA), a natural antioxidant and iron chelator, designed to combat oxidative stress in corneal epithelial injuries (Figure 5) [67]. The hydrogel demonstrated sustained PA release, preserved mitochondrial structure, and modulated oxidative stress markers, including COX2 and GPX4. These results support the potential of PVA hydrogels as customizable platforms for delivering redox-active therapies in wounds affected by oxidative imbalance.

**Figure 6:**
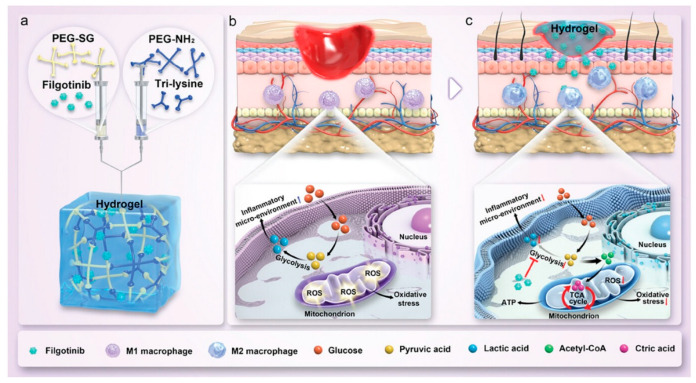
(**a**–**c**) Schematic illustration of the Fil@GEL hydrogel platform composed of PEG-SG, PEG-NH_2_, and tri-lysine, designed for sustained filgotinib delivery and wound healing [61]. Copyright © 2024, Wiley.

**Polyethylene glycol (PEG)** is a hydrophilic polymer with excellent biocompatibility and low immunogenicity, widely employed in hydrogel scaffolding and drug delivery systems [68,69]. Its flexible chemistry enables the formation of injectable, in-situ gelling systems with tunable mechanical strength and degradation profiles [70,71,72]. Xie et al. (2024) designed a PEG-based hydrogel (Fil@GEL) incorporating the JAK1 inhibitor filgotinib to modulate macrophage behavior in inflammatory wounds (Figure 6) [61]. Constructed from PEG-SG, PEG-NH_2_, and tri-lysine, the hydrogel formed rapidly upon injection, adhered strongly to tissue, and supported porosity for nutrient exchange. In vivo, Fil@GEL promoted anti-inflammatory (M2) macrophage polarization, reduced interleukin-1 beta (IL-1β) and interleukin-6 (IL-6) levels, and enhanced collagen and fibronectin deposition-demonstrating the utility of PEG hydrogels as platforms for immunometabolic intervention.

**Figure 7:**
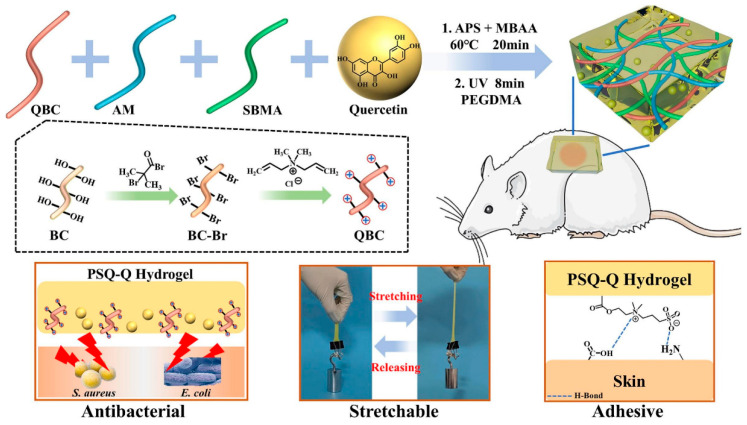
Schematic illustration of the PSQ-Q hydrogel composed of PAAm, SBMA, quercetin, and QBC, designed for infected wound healing [77]. Copyright © 2024, Elsevier.

**Polyacrylamide (PAAm)** is another synthetic polymer widely used in hydrogel networks for its high water content, softness, and strong crosslinked stability [73,74,75,76]. Particularly useful in mechanically dynamic or load-bearing wounds, PAAm can be integrated into double-network systems to enhance elasticity and functional performance. Rong et al. (2025) developed a multifunctional PAAm-based hydrogel by combining it with sulfobetaine methacrylate (SBMA), quercetin, and cationic bacterial cellulose (QBC) for managing *S. aureus*-infected wounds (Figure 7) [77]. The hydrogel exhibited excellent stretchability, substrate adhesion (~194.4 kPa), and biocompatibility. Its antibacterial, antioxidant, and anti-inflammatory properties synergistically accelerated wound healing, with a 98.9% closure rate by day 15 in animal models. This study underscores the capacity of PAAm systems to accommodate complex, multi-targeted wound interventions.

Synthetic polymer-based hydrogels expand the material design space in wound healing by enabling programmable architecture, sustained release capabilities, and structural resilience. While their biological inertness often necessitates the inclusion of functional additives, their chemical stability and reproducibility provide a strong foundation for engineering responsive and multifunctional wound dressings.

### 2.3. Limitations of Traditional Hydrogels in Wound Healing

Despite extensive research and clinical application, traditional hydrogel systems often face critical limitations when confronted with the biological complexity and variability of real-world wound environments. While these materials provide basic advantages such as moisture retention, biocompatibility, and ease of application, they frequently fall short in addressing the multifaceted challenges of wound healing, like microbial infection, oxidative stress, chronic inflammation, immune dysregulation, and impaired tissue regeneration, etc. [78]. These shortcomings are particularly apparent in non-healing wounds characterized by high exudate levels, irregular wound geometry, and prolonged inflammatory states. As wound healing is a dynamic, multi-phase process, effective dressings must integrate multifunctionality, adaptability, and long-term resilience, qualities not fully realized by most conventional hydrogels.

Natural polymer-based hydrogels, including those derived from hyaluronic acid, chitosan, alginate, and gelatin, are favored for their intrinsic biocompatibility, biodegradability, and biological signaling potential. However, these materials are often mechanically weak, prone to rapid enzymatic degradation, and exhibit poor adhesion under physiological conditions [79,80]. Inconsistencies in polymer source and extraction method can further lead to batch-to-batch variability, while their sensitivity to pH and enzymatic environments may compromise hydrogel performance in vivo [81]. Although chemical modification and blending strategies have been used to enhance structural robustness, these approaches can introduce manufacturing complexity, reduce reproducibility, and, at times, diminish the native bioactivity of the polymers. As a result, their application is often restricted to specific wound types and requires careful customization [82].

Synthetic polymer-based hydrogels, such as those formed from PVA, PEG, and PAAm, offer greater control over crosslinking architecture, mechanical strength, and degradation rate. Their consistency and scalability make them attractive for engineering reproducible dressing platforms [83]. However, these systems typically lack intrinsic biological signaling properties and require incorporation of exogenous bioactive components, such as peptides, growth factors, or antioxidants, to exert therapeutic effects [84]. Such modifications may increase production cost and regulatory burden while also introducing stability concerns. Moreover, some synthetic hydrogels rely on exogenous stimuli (e.g., temperature, light) for activation or drug release, which can complicate clinical translation in varied care settings. Long-term biodegradability, immune compatibility, and integration with regenerating tissue also remain ongoing concerns for purely synthetic systems. In addition to mechanical strength and bioactivity, the density of hydrogels critically influences their wound healing efficacy. A denser hydrogel matrix may enhance cellular adhesion and proliferation by providing more anchoring points while also modulating fluid absorption to better manage exudate levels. However, overly dense structures can impede nutrient diffusion and cell infiltration, highlighting the need for optimal balance.

In addition to their chemical and mechanical characteristics, hydrogel density has emerged as a critical factor in therapeutic performance. A denser matrix may improve cell adhesion and proliferation by offering more anchoring sites, and can also regulate fluid absorption, thereby aiding in exudate management [85]. However, excessive density may hinder nutrient diffusion and cell infiltration, undermining tissue integration and slowing wound resolution [86]. Thus, balancing polymer concentration and network porosity is essential to optimize the biological functionality of both natural and synthetic hydrogels [87].

Together, these limitations reflect the central challenge of hydrogel design: how to simultaneously achieve mechanical robustness, biological responsiveness, and clinical practicality. Traditional hydrogels typically succeed in one or two of these domains, but rarely across all. In response, growing attention has shifted toward nanohybrid hydrogels, which incorporate nanoscale components into polymeric networks to create multifunctional systems. These platforms combine the biological richness of natural polymers with the tunability of synthetic matrices, while leveraging the surface activity, controlled release capacity, and therapeutic versatility of nanomaterials. The next section explores the design principles, material architectures, and functional outcomes of these next-generation hydrogel systems.

### 2.4. Fabrication Techniques for Hydrogel-Based Wound Dressings: From Traditional Approaches to 3D Printing

The fabrication method plays a pivotal role in determining the structural integrity, drug-release kinetics, and biofunctionality of hydrogel-based wound dressings. Traditional techniques such as solvent casting, freeze–thaw cycling, and both ionic and chemical crosslinking remain widely used due to their simplicity and scalability. For instance, solvent casting involves drying polymer solutions into thin films [88], while freeze–thaw processes induce physical crosslinking through crystallization, commonly applied to PVA systems [89]. Ionic crosslinking, typically employed for alginate-based hydrogels, uses multivalent ions like Ca^2+^ to form gel matrices [90]. Chemical crosslinkers such as glutaraldehyde and genipin offer durable covalent bonding, particularly useful in synthetic hydrogels [91]. Although effective, these conventional methods offer limited control over spatial architecture and functional complexity

To overcome these limitations, emerging fabrication technologies such as 3D printing are increasingly applied to hydrogel systems for wound care. 3D printing enables precise, layer-by-layer deposition of hydrogel “bioinks” that may include nanomaterials, therapeutic agents, or living cells [92,93]. Recent advances include 3D-printed nanohybrid hydrogels with embedded antibacterial agents [94], angiogenic factors [95], or redox-active nanoparticles [96], resulting in accelerated healing and improved wound closure in preclinical models. Furthermore, smart hydrogels integrated into bioprinting platforms offer personalized wound care solutions that respond dynamically to the wound microenvironment. Despite these advances, challenges remain in optimizing print fidelity, cell viability, and material standardization

In parallel, techniques such as microfluidics [97], photolithography [98], and electrospinning [99] are also gaining traction. These methods allow for high-resolution control over hydrogel architecture, drug compartmentalization, and stimuli-responsive features. Critically, they are highly compatible with nanohybrid designs, facilitating the incorporation of multifunctional nanoparticles and enabling spatiotemporal therapeutic delivery. As fabrication techniques become more sophisticated, their integration with nanomaterial engineering is expected to drive the development of next-generation, intelligent hydrogel dressings tailored for dynamic and complex wound environments

## 3. Emergence of Nanohybrid Hydrogels

### 3.1. Definition and Design Principles

Nanohybrid hydrogels offer a versatile platform to address key biological barriers in wound healing that traditional hydrogels often fail to resolve. By embedding nanomaterials within polymeric matrices, these systems introduce new functionalities such as antibacterial activity, redox regulation, immunomodulation, angiogenic stimulation, and responsiveness to internal or external cues. Each of these capabilities corresponds to a phase or challenge in the wound healing process, making nanohybrid hydrogels uniquely equipped to address complex or chronic wound scenarios [100,101,102].

### 3.2. Functional Advantages in Wound Healing

#### 3.2.1. Antibacterial Activity

Infection remains a major impediment to effective wound healing, as microbial colonization can disrupt normal inflammatory resolution and delay tissue regeneration. Traditional hydrogels often lack intrinsic antibacterial function and rely on passive delivery of antimicrobial agents, which can be insufficient or promote resistance. Nanohybrid hydrogels, by contrast, incorporate antibacterial nanostructures such as metal nanoparticles or catalytic nanozymes that actively suppress microbial growth through multiple mechanisms—including ion release, membrane disruption, and ROS generation [103,104,105,106,107,108,109].

**Figure 8:**
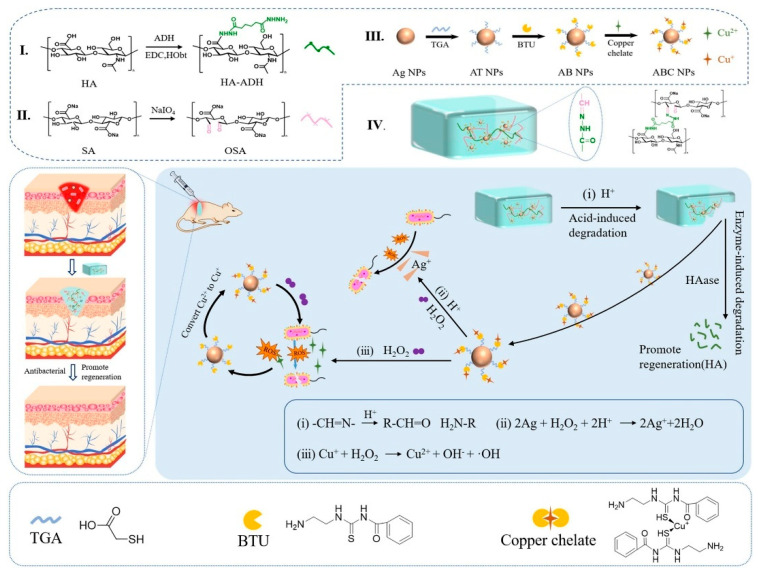
Schematic representation of the construction and therapeutic processes of HS@ABC hydrogel [110]. Copyright © 2025, Elsevier.

Li et al. (2025) developed an injectable nanohybrid hydrogel (HS@ABC) incorporating silver–copper bimetallic nanozymes within a hyaluronic acid–sodium alginate matrix (Figure 8) [110]. This formulation exerted a synergistic antimicrobial effect through Ag^+^ and Cu^2+^ ion release and chemodynamic therapy (CDT), achieving over 99.9% inhibition of *Staphylococcus aureus* (*S. aureus*) and *Escherichia coli* (*E. coli*) in vitro. Importantly, the hydrogel actively modulated the immune response during the inflammatory and proliferative phases of wound healing. In a murine infected wound model, treatment with the nanohybrid hydrogel led to significant reductions in pro-inflammatory cytokines IL-6 and tumor necrosis factor-alpha (TNF-α), while upregulating anti-inflammatory interleukin-10 (IL-10), compared to both untreated and blank hydrogel controls. This cytokine profile shift promoted faster resolution of inflammation, enhanced granulation tissue formation, and accelerated re-epithelialization. Relative to normal healing dynamics, the hydrogel-treated wounds exhibited a shortened inflammatory phase and earlier onset of tissue remodeling. These findings underscore the potential of immunomodulatory nanohybrid hydrogels to guide wound healing progression in infection-prone environments.

#### 3.2.2. Antioxidant and ROS Scavenging

Oxidative stress is a critical barrier to wound healing, particularly in chronic wounds where excessive ROS impair keratinocyte migration, fibroblast proliferation, and angiogenesis [111,112]. Conventional hydrogels lack dynamic antioxidant properties and often depend on incorporated antioxidants with short half-lives or limited release control. Nanohybrid hydrogels address this limitation by incorporating ROS-scavenging nanomaterials such as cerium oxide, polydopamine, or enzyme-mimetic nanozymes that offer sustained redox modulation within the wound microenvironment [113,114,115].

**Figure 9:**
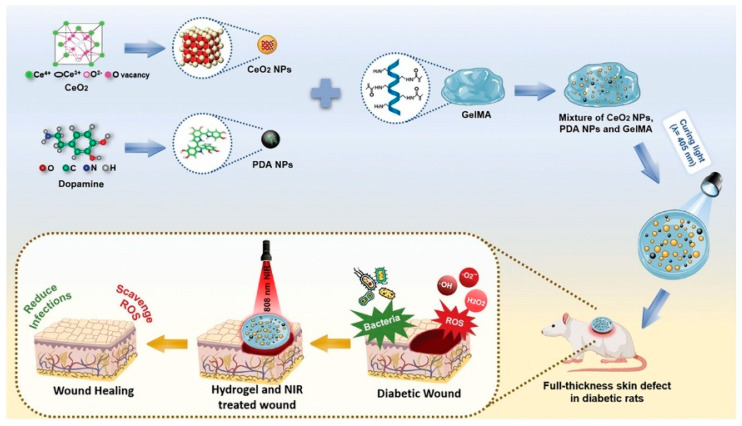
Schematic of NIR-responsive GelMA/CeO_2_/PDA hydrogel design and preparation, along with its effects on diabetic wound healing [116]. Copyright © 2025, Wiley.

Xue et al. (2025) developed a multifunctional nanohybrid hydrogel (GelMA/CeO2/PDA) by integrating cerium oxide nanoparticles (CeO_2_) and polydopamine (PDA) into a GelMA matrix, enabling both ROS scavenging and near-infrared (NIR)-responsive photothermal activity (Figure 9) [116]. CeO_2_ neutralized ROS via redox cycling between Ce^3+^ and Ce^4+^, while PDA enhanced photothermal activation and hydrogel cohesion. In a diabetic wound model, the hydrogel not only suppressed oxidative stress but also reshaped the immune microenvironment during the inflammatory and proliferative phases. Notably, it downregulated IL-6, interleukin-17 (IL-17), and matrix metalloproteinase-9 (MMP9), which are linked to chronic inflammation and matrix degradation, while upregulating TGF-β, vascular endothelial growth factor (VEGF), alpha-smooth muscle sctin (α-SMA), and activator protein-1 (AP-1)—markers associated with angiogenesis, fibroblast activation, and tissue remodeling. Compared to untreated diabetic wounds, which often stall at the inflammatory phase, the hydrogel promoted earlier vascularization and granulation tissue formation. This study illustrates how redox-active nanohybrids can simultaneously regulate oxidative and immune signals to re-establish a regenerative wound healing trajectory.

#### 3.2.3. Angiogenesis Promotion

Neovascularization is essential for tissue regeneration, as it enables oxygen and nutrient delivery to healing tissue [117]. Yet in ischemic or diabetic wounds, angiogenesis is often impaired by inflammation and oxidative stress [118]. Traditional hydrogels lack the capacity to orchestrate angiogenic signaling or protect labile growth factors. Nanohybrid hydrogels provide a solution by co-delivering pro-angiogenic factors with protective nanocarriers or by creating redox-modulating environments that indirectly support endothelial cell function [17,119].

**Figure 10:**
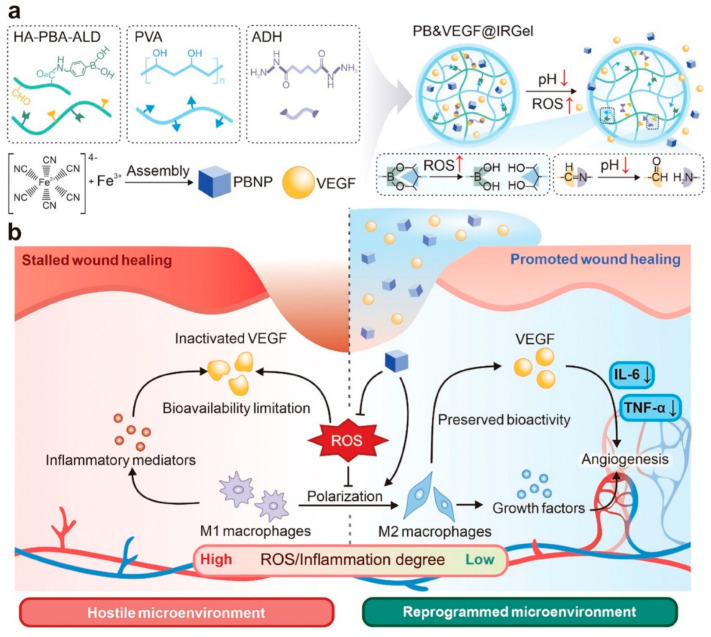
(**a**,**b**) Schematic of PB&VEGF@IRGel for diabetic wound healing [120]. Copyright © 2024, Wiley.

He et al. (2024) engineered an inflammation-responsive nanohybrid hydrogel (PB&VEGF@IRGel) by embedding VEGF and Prussian Blue nanoparticles (PBNPs) into a hydrogel matrix that reacts to oxidative stress (Figure 10) [120]. In a diabetic wound model, this hydrogel responded to high ROS levels by scavenging oxidative species through PBNPs while preserving the angiogenic activity of VEGF. The combined action significantly promoted wound healing: the PB&VEGF@IRGel group exhibited nearly complete wound closure by day 14, with accelerated re-epithelialization, thicker epidermis, and enhanced collagen I deposition. Immunofluorescence analysis confirmed a substantial increase in cluster of differentiation 31 (CD31)-positive vessels, and transcriptomic analysis revealed upregulation of angiogenesis-related genes including VEGFA and angiopoietin-2 (ANGPT2), alongside suppression of inflammatory mediators such as IL-6 and TNF-α. The hydrogel also promoted macrophage polarization toward the M2 phenotype (CD206, ARG1), helping resolve inflammation. Compared to untreated diabetic wounds, which showed prolonged inflammation and poor neovascularization, PB&VEGF@IRGel created a pro-regenerative microenvironment. This study underscores the potential of ROS-responsive hydrogels to coordinate immunomodulation and vascular regeneration in chronic wounds.

#### 3.2.4. Immune Modulation and Anti-Inflammatory Effects

A balanced immune response is vital for timely wound resolution [121,122]. Chronic wounds are often characterized by prolonged pro-inflammatory (M1) macrophage polarization and excessive inflammatory cytokine production. Nanohybrid hydrogels offer a localized and tunable strategy for immune modulation by integrating photothermal agents, redox regulators, or cytokine-mimicking nanomaterials into the hydrogel matrix [123,124].

**Figure 11:**
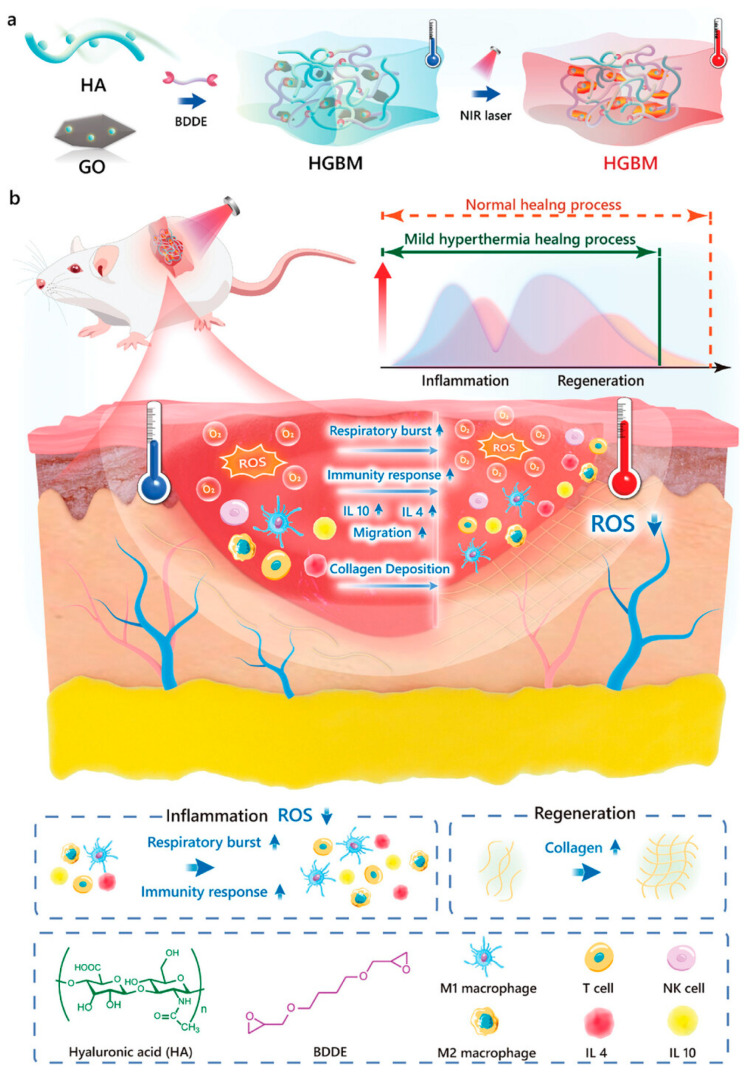
(**a**,**b**) Schematic illustration of the fabrication and photothermal effect of HGBM hydrogel as a mild thermal stimulation device [125]. Copyright © 2024, Wiley.

Zhang et al. (2024) developed a graphene oxide-reinforced hyaluronic acid hydrogel (HGBM) that leverages NIR photothermal stimulation to actively modulate immune processes during wound healing (Figure 11) [125]. Under mild hyperthermia (~45 °C) triggered by NIR exposure, the hydrogel induced a transient elevation of ROS, accelerating early immune cell infiltration and initiating a respiratory burst at the wound site. This was followed by a timely resolution of inflammation marked by a higher M2-to-M1 macrophage ratio, evidenced by increased cluster of differentiation 206 (CD206) and decreased inducible nitric oxide synthase (iNOS) expression. In a full-thickness wound model, this immune reprogramming correlated with enhanced re-epithelialization, elevated collagen I deposition, and improved blood perfusion by day 7. Ribonucleic acid (RNA) sequencing further revealed significant upregulation of immunoregulatory genes, including interleukin-4 (IL-4) and IL-10, alongside downregulation of hypoxia-inducible factor-1 (HIF-1) and p53 pathway genes—suggesting a reduction in hypoxic stress. Compared to untreated wounds, which showed delayed immune resolution, HGBM facilitated a faster transition from inflammation to tissue regeneration. This study exemplifies how thermally responsive nanohybrids can precisely orchestrate immune dynamics for accelerated repair in chronic wound environments.

#### 3.2.5. Stimuli-Responsive Behavior

Responsive hydrogels can adapt to local wound conditions or external stimuli to control drug release and therapeutic activation [12,13,14]. Such behavior is particularly useful in chronic or non-healing wounds where static dressing properties are insufficient. Nanohybrid systems combine nanomaterials with thermoresponsive, pH-sensitive, or photoactive polymers to achieve spatial and temporal precision.

**Figure 12:**
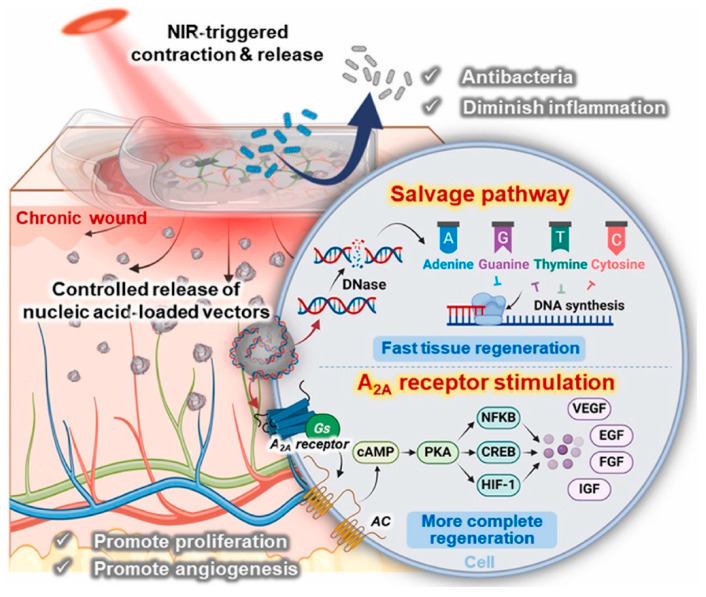
A MoS_2_ nanosheets-mediated NIR-responsive hydrogel loaded with polydeoxyribonucleotide (PDRN) nano-vectors is proposed for fast and complete chronic wound healing [126]. Copyright © 2025, Elsevier.

Sun et al. (2025) developed a thermoresponsive nanohybrid hydrogel (PCNPs@NIR-gel) by embedding MoS_2_ nanosheets and polynucleotide (PDRN)-loaded nanovectors into a PNIPAAm–chitosan matrix (Figure 12) [126]. Upon NIR exposure, MoS_2_ enabled localized photothermal heating, triggering gel contraction and precise PDRN release. In a diabetic wound model, this stimuli-responsive system promoted regeneration during the inflammatory and proliferative phases, significantly reducing wound area by day 14. Histological evaluation showed enhanced re-epithelialization, collagen deposition, and a narrowed granulation tissue gap. Immunohistochemistry confirmed increased expression of VEGF, α-SMA, and TGF-β, alongside reduced MPO (myeloperoxidase), indicating angiogenesis promotion and inflammation suppression. Transcriptomic profiling further revealed activation of key angiogenic and immunomodulatory pathways, supported by enriched Kyoto Encyclopedia of Genes and Genomes (KEGG) and gene ontology (GO) terms. These findings demonstrate how NIR-triggered hydrogels can deliver synchronized therapeutic cues to dynamically match the wound microenvironment and improve healing outcomes in chronic wounds.

### 3.3. Representative Nanomaterials in Nanohybrid Hydrogels

#### 3.3.1. Metal-Based Nanoparticles

Metal-based nanoparticles are widely employed in nanohybrid hydrogels for their multifunctional properties, including antimicrobial activity, ROS modulation, and structural crosslinking [127]. Among them, ions such as Ag^+^, Cu^2+^, and Zn^2+^ are frequently selected for their redox activity and ability to coordinate with functional groups in polysaccharides or peptides, facilitating both therapeutic and architectural roles [128,129,130]. For instance, Zn^2+^ can serve as a crosslinker that induces nanofibrillar self-assembly while simultaneously promoting angiogenesis and immune modulation. This dual function was elegantly demonstrated in a konjac glucomannan-based hydrogel system, where Zn^2+^ triggered the assembly of glycyrrhizic acid into a nanofiber network while reinforcing a photo-crosslinked polymer matrix [131]. Similar hybrid constructs have been explored with silver and copper systems, leveraging the ions’ catalytic and bactericidal mechanisms [132,133]. Overall, metal-based nanomaterials offer a unique combination of bioactivity and network-forming potential, making them a cornerstone in the design of structurally robust and biologically active nanohybrid hydrogels.

#### 3.3.2. Metal Oxide Nanozymes

Metal oxide nanozymes, including CeO_2_, MnO_2_, and Fe_3_O_4_, are widely used in nanohybrid hydrogels due to their catalytic mimicry of antioxidant enzymes [134]. These nanoparticles can decompose hydrogen peroxide, scavenge superoxide, and regulate local redox balance-functions critical for managing oxidative stress in chronic wounds [135,136,137]. Unlike traditional antioxidants, nanozymes are catalytically regenerative, offering sustained activity under inflammatory or hypoxic conditions.

MnO_2_, in particular, is frequently integrated into adhesive or injectable hydrogels, where it contributes to both ROS modulation and oxygen generation [138,139]. When coupled with polymers such as hyaluronic acid or dopamine-modified biogels, MnO_2_ can synergistically support angiogenesis, macrophage polarization, and infection resolution [140]. Its dual function as both a biochemical modulator and structural crosslinker makes metal oxide nanozymes a versatile platform for redox-responsive hydrogel design.

#### 3.3.3. Carbon-Based Nanomaterials

Carbon-based nanomaterials, including graphene oxide (GO), reduced graphene oxide (rGO), carbon dots, and fullerenes, are widely employed in nanohybrid hydrogels due to their unique physicochemical versatility. These materials offer high surface area, π-conjugated structures, tunable conductivity, and inherent photothermal or antioxidant properties [141,142,143,144]. When incorporated into hydrogel matrices, they can enhance structural integrity, mediate controlled drug release, and contribute photothermal or photodynamic effects under external stimulation.

For example, reduced graphene oxide and glycine-modified fullerene have been co-integrated into smart hydrogel systems to provide pH/glucose-responsive drug release alongside NIR-triggered antibacterial and anti-inflammatory effects [145]. In such platforms, rGO contributes thermal conductivity and near-infrared responsiveness, while fullerenes scavenge ROS and support cellular redox balance. These multi-functional carbon nanofillers not only improve wound healing kinetics in diabetic models but also enable the design of stimuli-responsive hydrogels with tightly regulated bioactivity. Their modular surface chemistry and compatibility with soft polymeric scaffolds continue to make carbon-based nanomaterials central to advanced hydrogel engineering.

#### 3.3.4. Polymeric Nanoparticles and Nanogels

**Figure 13:**
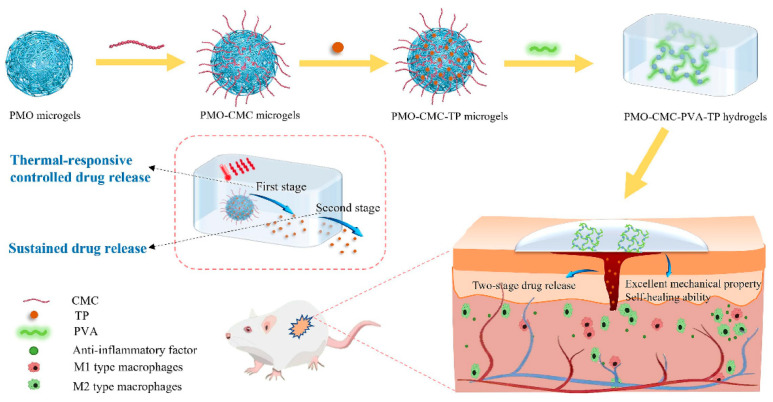
The fabrication of PMO-CMC-PVA-TP hydrogels and their treatment of chronic wound [148]. Copyright © 2025, Elsevier.

Polymeric nanogels offer a unique advantage in wound healing due to their soft, tunable structure, high drug loading capacity, and environmental responsiveness. Their nanoscale size allows for efficient drug encapsulation and controlled release, while their chemical versatility enables integration into hydrogel networks without compromising biocompatibility [146,147]. A recent study by Liu et al. (2025) illustrates this potential through the development of a thermal-responsive nanohybrid hydrogel system composed of PMO-CMC nanogels embedded within a polyvinyl alcohol (PVA)-borax matrix for chronic wound treatment (Figure 13) [148]. The poly(ethylene glycol)-based nanogels were modified with carboxymethyl cellulose (CMC) to enhance hydrophilicity, mechanical reinforcement, and drug loading. This system exhibited a two-stage drug release behavior: rapid, temperature-triggered delivery from the nanogels and sustained diffusion from the PVA matrix. Incorporating the nanogels improved the self-healing properties and mechanical integrity of the hydrogel, while also enabling a responsive release of tea polyphenols (TP), an anti-inflammatory agent. In vivo, the TP-loaded PMO-CMC-PVA hydrogels significantly accelerated wound closure, improved histological outcomes, and reduced inflammation in a chronic wound mouse model. This study highlights the capacity of polymeric nanogels to endow hydrogels with finely tunable release kinetics, enhanced structural performance, and therapeutic precision, marking them as valuable components in next-generation wound dressings.

#### 3.3.5. Emerging Hybrid Nanostructures

Emerging nanostructures such as metal-organic frameworks (MOFs), MXenes, and Prussian Blue analogs have gained increasing attention in nanohybrid hydrogel engineering due to their customizable architecture, high surface area, and multifunctionality [149,150,151]. Among these, MOFs offer distinct advantages as both therapeutic carriers and catalytic nanozymes. Their porous crystalline structure enables efficient loading of small molecules, while their tunable metal centers and organic linkers allow for environment-responsive behavior, making them ideal candidates for treating complex, chronic wounds [152].

For instance, titanium-based MOFs (NH_2_-MIL-125(Ti)) have been incorporated into phenylboronate-crosslinked hydrogels to form smart, adhesive wound dressings responsive to acidic and oxidative environments [153]. Under visible light, these MOFs generate hydroxyl radicals that exhibit potent antibacterial activity, while concurrently releasing anti-inflammatory agents such as metformin. The resulting hydrogel matrix demonstrates tissue adhesion, self-healing, and mechanical adaptability, along with accelerated diabetic wound healing through enhanced angiogenesis and immune modulation. Such multifunctional hybrid systems exemplify the potential of MOFs to serve as active therapeutic platforms within hydrogels, enabling synchronized control of oxidative stress, infection, and tissue regeneration.

### 3.4. Summary of Representative Nanomaterials

The integration of nanomaterials into hydrogel matrices has fundamentally reshaped the performance landscape of wound dressings, offering multifunctional capabilities that conventional systems lack. Each class of nanomaterials brings distinct advantages to hydrogel composites, from the broad-spectrum antibacterial properties of metal nanoparticles to the ROS-scavenging abilities of nanozymes, and the intelligent, stimulus-responsive behaviors enabled by carbon-based or polymeric nanostructures. These combinations have been purposefully engineered to address core therapeutic challenges such as infection, oxidative stress, chronic inflammation, impaired angiogenesis, and inefficient drug delivery.

To contextualize the breadth of innovation in this area, Table 1 summarizes representative studies across five primary nanomaterial categories, highlighting their structural components, functions, wound models, and citation references. This overview illustrates the diverse design strategies employed in recent nanohybrid hydrogel platforms and underscores their translational potential across multiple wound types, including diabetic, burn, and infected wounds.

## 4. Research Prospects

Despite significant advances in the development of nanohybrid hydrogels for wound healing, several critical challenges must be addressed to translate laboratory innovations into clinically effective therapies. Looking ahead, the field is poised to benefit from more rational design approaches, interdisciplinary integration, and regulatory foresight. Below, we outline key areas where future research can meaningfully advance the clinical potential of nanohybrid hydrogel systems.

### 4.1. Rational Material Design and Standardization

A major limitation of current hydrogel development lies in the empirical and fragmented nature of material formulation. Most nanohybrid systems are optimized through trial-and-error methods, resulting in high variability and poor reproducibility across different laboratories [168,169]. Future efforts should focus on computational material modeling, machine learning-guided nanocomposite design, and the use of standardized synthesis and testing protocols. These strategies can accelerate the identification of optimal nanomaterial-hydrogel combinations and facilitate comparison across studies. Moreover, high-throughput screening platforms and predictive in silico tools could enable rapid prototyping of multifunctional hydrogels tailored to specific wound types.

### 4.2. Bioactivity-Biocompatibility Trade-Offs

While nanomaterials such as metal nanoparticles, nanozymes, and carbon-based structures offer enhanced biofunctionality, including antibacterial, antioxidant, and angiogenic effects, they also introduce new biocompatibility concerns. Some nanomaterials may exhibit dose-dependent cytotoxicity, unintended immune activation, or problematic degradation byproducts [170,171]. Future research must prioritize the systematic evaluation of long-term biosafety, particularly in chronic wound settings where prolonged hydrogel exposure is common. Strategies such as biodegradable nanocarriers, surface passivation, and responsive clearance mechanisms may help reconcile the trade-off between bioactivity and biocompatibility.

### 4.3. Smart, Responsive, and Feedback-Controlled Systems

One of the most transformative directions for nanohybrid hydrogels lies in the evolution from passive carriers to intelligent, dynamically responsive systems. While many current designs respond to local stimuli such as pH, ROS, or glucose, the next generation of materials will incorporate feedback-controlled release, self-adjusting mechanical properties, and even biosignal-responsive behavior. Such systems could autonomously modulate drug delivery in response to real-time wound status, mimicking physiological healing processes. Embedding microelectronic sensors or wearable interfaces into hydrogel matrices could enable closed-loop therapeutic platforms that deliver personalized wound care [172].

### 4.4. Clinical Translation and Regulatory Pathways

Although numerous nanohybrid hydrogel formulations have demonstrated impressive therapeutic efficacy in preclinical models, only a small fraction have progressed toward human trials. This translational gap is driven by challenges in large-scale production, batch consistency, sterilization, shelf-life extension, and cost-effectiveness [173,174]. Regulatory uncertainty, particularly regarding how nanohybrid materials are classified (as a drug, device, or combination product), also complicates commercialization [175]. Collaboration with regulatory bodies, early-stage clinical validation, and the development of good manufacturing practice (GMP)-compliant synthesis routes will be essential for de-risking and accelerating clinical translation.

### 4.5. Interdisciplinary Integration and Emerging Technologies

In the context of personalized therapeutics, platelet-derived biomaterials such as platelet-rich plasma (PRP) and platelet lysates represent endogenous reservoirs of regenerative cues tailored to individual patients [176]. These preparations are rich in growth factors—including platelet-derived growth factor (PDGF), VEGF, and TGF-β—that accelerate tissue regeneration [176]. However, limitations such as rapid degradation and burst release profiles hinder their sustained therapeutic efficacy [177]. The integration of platelet-derived components into nanohybrid hydrogel matrices provides a promising strategy for controlled, localized release while maintaining structural support and immunomodulatory balance [178,179]. This synergistic combination enhances wound healing dynamics and aligns with the emerging paradigm of bioresponsive, patient-specific therapy. Looking ahead, advanced hydrogel systems may incorporate programmable release kinetics tailored to an individual’s platelet profile, enabling on-demand regenerative and immune responses.

More broadly, the future trajectory of nanohybrid hydrogel systems will be shaped by interdisciplinary convergence. Technologies such as 3D bioprinting can enable spatially defined, patient-specific hydrogel constructs, while artificial intelligence (AI) can assist in optimizing formulation design and predicting healing outcomes. Integration with soft electronics, biosensors, and nanovalve-controlled delivery mechanisms will further transform hydrogels from passive dressings into interactive, responsive wound care platforms. The convergence of materials science, synthetic biology, digital health, and regenerative medicine heralds a new era of intelligent wound healing systems with unprecedented therapeutic potential [172].

## 5. Conclusions

Over the past decade, nanohybrid hydrogels have emerged as a transformative class of biomaterials in the field of wound healing, offering integrated solutions to challenges that traditional hydrogel systems alone cannot address. By incorporating nanoscale components into polymeric hydrogel networks, these systems unlock multifunctional capabilities-such as antimicrobial activity, reactive oxygen species scavenging, angiogenesis promotion, and immune modulation while retaining the biocompatibility and structural advantages of conventional hydrogel platforms.

This review has highlighted both the progress and complexity of nanohybrid hydrogel design, with detailed examination of natural and synthetic polymer matrices, diverse classes of functional nanomaterials, and their synergistic mechanisms of action across infected, diabetic, and chronic wound models. The representative studies demonstrate a clear trajectory toward smarter, more responsive, and bioactive dressing platforms that are tailored to the dynamic and multifactorial nature of wound environments.

Looking ahead, the clinical translation of these systems will depend on interdisciplinary collaboration, scalable manufacturing, and regulatory innovation. Future efforts must prioritize safety, reproducibility, and integration with emerging technologies such as biosensing, AI-driven design, and tissue-engineered scaffolds to fully realize the potential of nanohybrid hydrogels in precision wound care. As the field moves forward, these advanced materials stand poised to redefine how we understand and treat complex wounds in both acute and chronic settings.

## Figures and Tables

**Table 1:** Representative nanomaterials incorporated into nanohybrid hydrogels for wound healing applications.

Material Category	Nanomaterial Type	Hydrogel Matrix	Function	Wound Model	Refs.
Metal-Based Nanoparticles	Silver clusters (AgC)	PHEMA-co-AAm (poly(hydroxyethyl methacrylate)-co-acrylamide)	Antibacterial, fibroblast alignment, moisture retention, and pattern-enhanced cell migration	In vitro scratch assay (MRC-5 fibroblasts)	[154]
	Cu^2+^ ions	Gelatin/Alginate (GA) + Propranolol	Antibacterial, angiogenesis promotion, sympathetic nerve modulation via β-receptor blockade	Infected full-thickness third-degree burn (SD rats)	[155]
	ZnO Nanostructures	CMC)/ Agarose / PVP (CAP1@ZnO)	Antibacterial, enhanced porosity, swelling, biocompatibility, and in vivo wound regeneration	Full-thickness wound model in BALB/c nude mice	[156]
	Gold nanoparticles (Au@MOFs)	Silk fibroin (SF) hydrogel	Synergistic photothermal and photodynamic antibacterial therapy; enhanced ROS generation; accelerated wound healing	MRSA-infected full-thickness rat skin wound	[157]
Metal Oxide Nanozymes	CeO_2_ nanoparticles	CMC)/ PVA foam-based hydrogel	Antibacterial activity, controlled drug release (silver sulfadiazine), high swelling ratio, biodegradable	In vitro antibacterial assay (*E. coli*, *S. aureus*); no animal model used	[158]
	MnO_2_ nanozymes + ε-Polylysine (E-PL)	Oxidized Gellan Gum (O-GG) + HA	Antibacterial, Antioxidant, Angiogenesis promotion, Injectability, Adhesion	Diabetic full-thickness wound in rats	[140]
	Iron-doped Carbon Dots (Fe-CDs)	Dual-crosslinked hydrogel based on Sodium Alginate (SA), Oxidized SA, and Carboxymethyl Chitosan (CMCS)	Peroxidase-like (POD) catalytic activity, antibacterial via Fenton reaction, anti-inflammatory, collagen deposition, and vascularization	Infected full-thickness skin wound model in mice	[159]
	Cu-doped Carbon Dots (CuCD1)	Carboxymethyl chitosan (CMCS) + Oxidized dextran (ODEX)	Biofilm penetration and eradication, ROS generation (POD-like), bacterial DNA damage	S. aureus-infected full-thickness skin wound model (Balb/c mice)	[160]
Carbon-Based Nanomaterials	Polydopamine-reduced graphene oxide compounded glycine-modified fullerene (GPC)	Dual dynamic Schiff base/phenylboronate ester crosslinked hyaluronic acid-chondroitin sulfate (AHP/OCS)	Synergistic photothermal/photodynamic antibacterial, pH/glucose-responsive drug release, anti-inflammatory, pro-angiogenesis, antioxidant	Diabetic foot ulcer (DFU) infected rat model	[145]
	Carbon Dots (CDs) + Ag_3_PO_4_	PAAm	Antibacterial (photodynamic and cationic synergistic), improved mechanical skin conformity	In vitro only (*E. coli*, *S. aureus*, *C. albicans*)	[161]
	Multi-walled carbon nanotubes (CNTs)	Alginate dialdehyde, PEI, PNIPAM	NIR-II-responsive drug release, multi-stimuli responsiveness (pH, H_2_O_2_, temp), antibacterial activity	In vitro, ex vivo (infected porcine skin), in vivo (rat model, spinal site)	[162]
Polymeric Nanoparticl-es and Nanogels	iRGD&PS-modified PLGA NPs loaded with Tiliroside	PVA/Gelatin	Promotes efferocytosis, macrophage reprogramming, angiogenesis; anti-inflammatory and ROS scavenging	Diabetic full-thickness skin wound (mouse model)	[163]
	Chitosan nanoparticles	SA hydrogel	Dual-drug delivery (Ciprofloxacin + Insulin), antibacterial, anti-inflammatory, cell proliferation and migration support	In vitro (L929 fibroblast scratch assay)	[164]
	Polydopamine (mPDA) nanogels + DFO@LA	Dopamine-hyaluronic acid (DA-HA)	Anti-inflammation, angiogenesis promotion, NIR-triggered on-demand drug release	Full-thickness skin defect in mice	[165]
MOFs	ZIF-8	SA/CMC/PVA (crosslinked with TEOS)	pH-responsive swelling, antibacterial, biocompatible, enhanced fibroblast adhesion and proliferation	In vitro only (3T3 fibroblast cell lines)	[166]
	HKUST-1 (Cu-based)	Chitosan/PVA composite hydrogel	Antibacterial, pro-angiogenic (Cu^2+^ release), granulation, collagen deposition	Full-thickness infected wound model in rats	[167]

## Data Availability

No new data were created or analyzed in this study. Data sharing is not applicable to this article.
